# Supplementary material for: Hypoxia Regulates Brown Adipocyte Differentiation and Stimulates miR-210 by HIF-1α
Source: Int J Mol Sci. 2024 Dec 26;26(1):117. doi: 10.3390/ijms26010117 (PMC11720532; doi:10.3390/ijms26010117)

Supplemental Figure S1. Immunofluorescence Images Submission.

To ensure transparency and reproducibility, all original, unmodified fluorescence images of cells used in the figures are provided here.

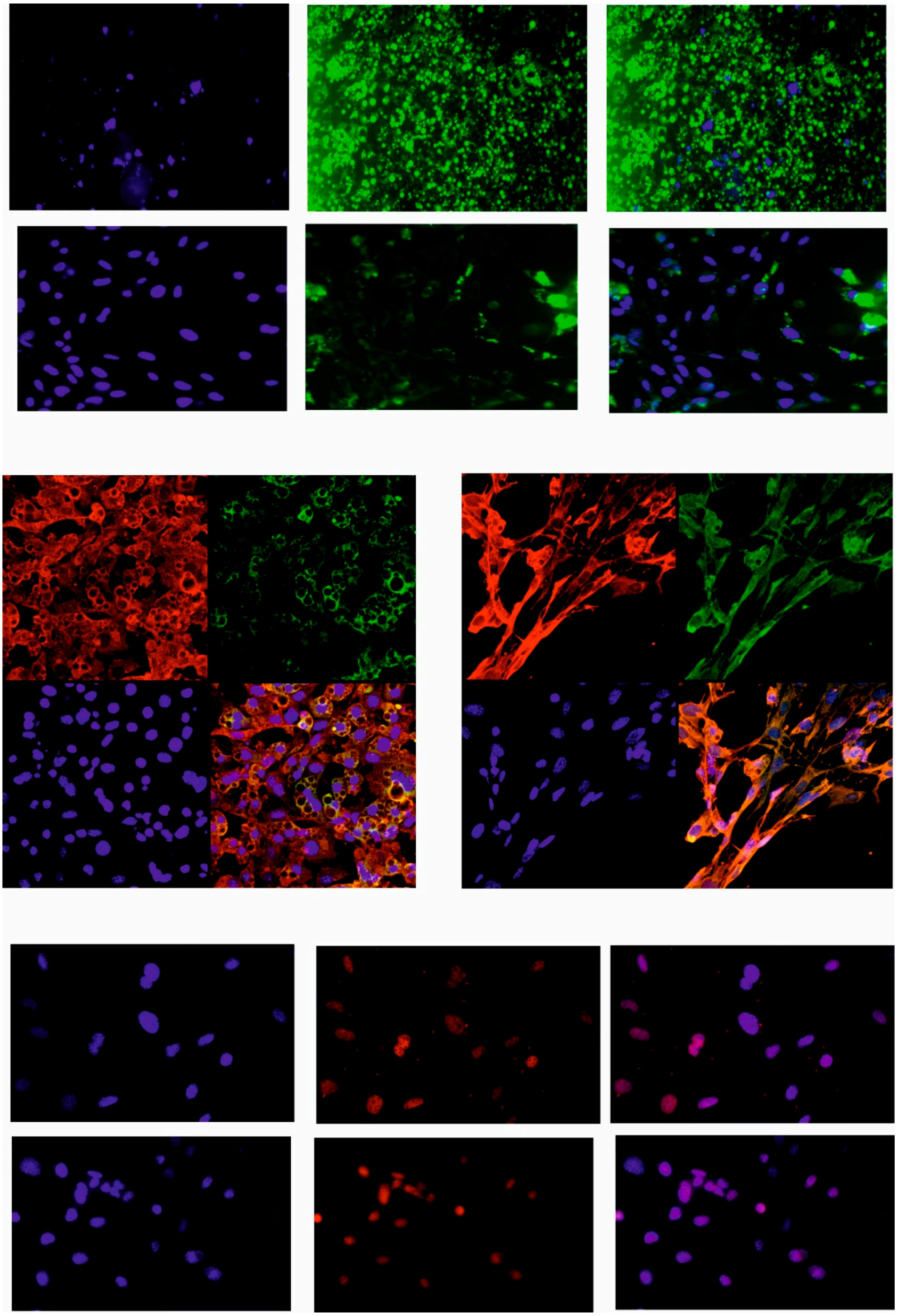

Supplemental Figure S2. Whole Blot Images Submission.

These whole blot images include all lanes, bands, and areas cropped for use in the main figures.

Fig.1

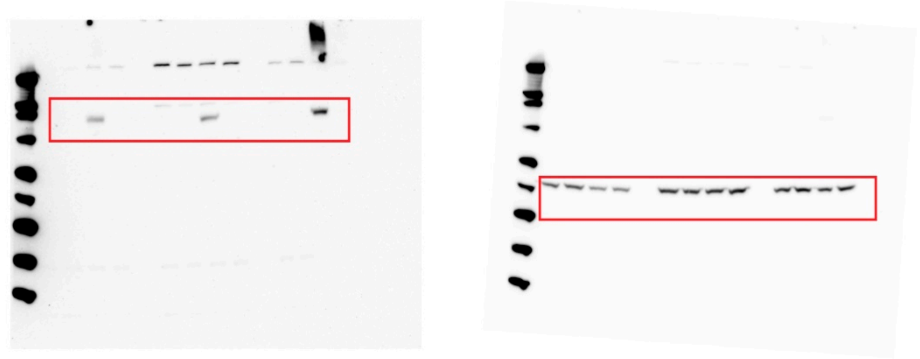

Fig. 2.

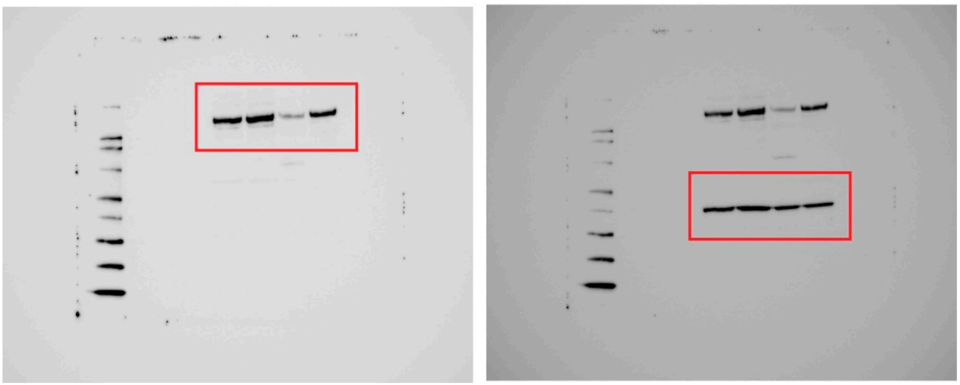

Fig.4

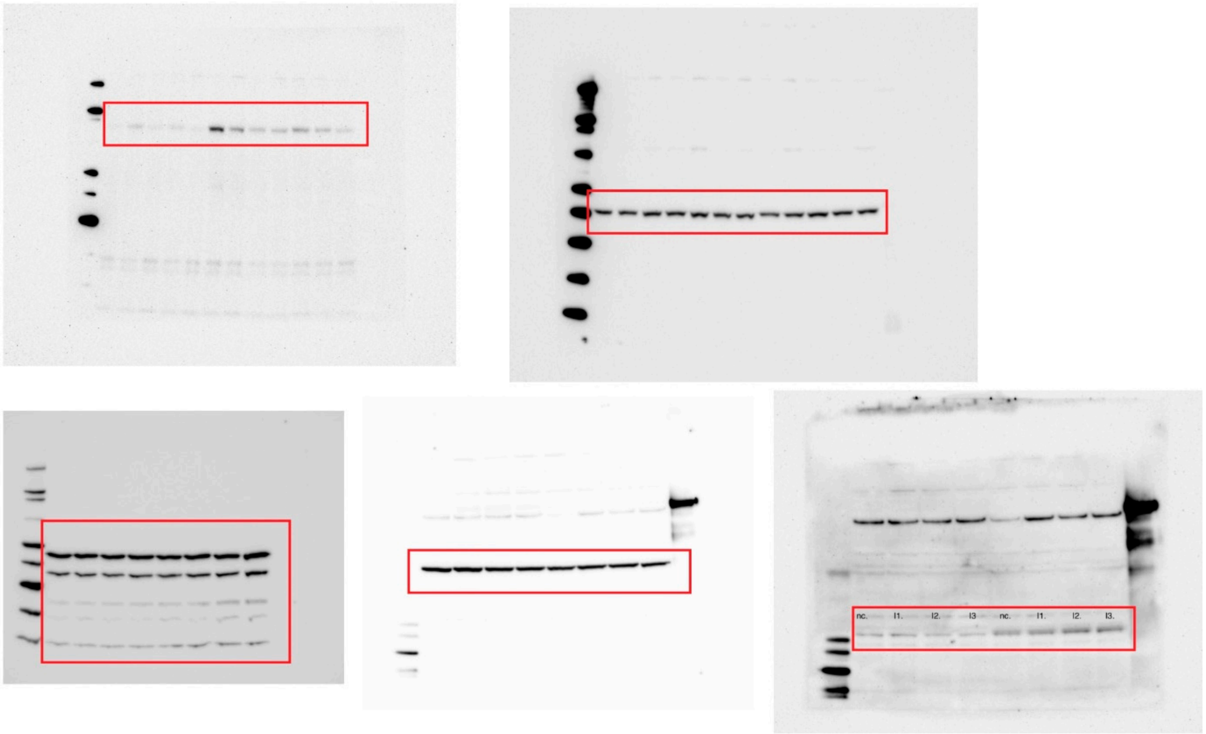

Supplement: Supplementary file 1 [file ijms-26-00117-s001.zip › ijms-3384400---supplementary figure.pdf]
