# Supplementary material for: Hypoxia Regulates Brown Adipocyte Differentiation and Stimulates miR-210 by HIF-1α
Source: Int J Mol Sci. 2024 Dec 26;26(1):117. doi: 10.3390/ijms26010117 (PMC11720532; doi:10.3390/ijms26010117)
Supplement: Supplementary file 1 [file ijms-26-00117-s001.zip › ijms-3384400-supplementary table.pdf]

Supplemental Table S1. Sequence of miRNA inhibitor and siRNA.

Gene	Supplier	Sequence (5′)
miR-210-5p Inhibitor	Qiagen	AGTGTGCGGTGGGCAG
Hif1a siRNA	Dharmacon	- UUUAAUACCCUCCGAUUUA<br>- UUACUGAGUUGAUGGGUUA<br>- GGAAAGAGAGUCAUAGAAC<br>- UGAGAGAAAUGCUUACACA

Supplemental Table S2. List of primers used for quantitative real-time PCR.

Gene	Forward primer (5′)	Reverse primer (5′)
Adipoq	GGAGAGAAAGGAGATGCAGGT	CTTTCCTGCCAGGGGTTC
Cd36	TTGTACCTATACTGTGGCTAAATGAGA	CTTGTGTTTTGAACATTTCTGCTT
Egln3	GTTTGGCTCCCTACCTTGTT	GGATGTCTGCAGGTGTTTCT
Elovl3	TTCTCACGCGGGTTAAAAATGG	GAGCAACAGATAGACGACCAC
Fabp4	GGATGGAAAGTCGACCACAA	TGGAAGTCACGCCTTTCATA
Pparg	TCGCTGATGCACTGCCTATG	GAGAGGTCCACAGAGCTGATT
Ppargc1a	TTCATCTGAGTATGGAGTCGCT	GGGGGTGAAACCACTTTTGTAA
Serpine1	TCTGGGAAAGGGTTCACTTTACC	GACACGCCATAGGGAGAGAAG
Tbp	AGAACAATCCAGACTAGCAGCA	GGGAACTTCACATCACAGCTC
Ucp1	AGGCTTCCAGTACCATTAGGT	CTGAGTGAGGCAAAGCTGATTT
Vegfa	AAAAACGAAAGCGCAAGAAA	TTTCTCCGCTCTGAACAAGG
miR-210-3p	hsa-miR-210-3p (YP00204333)<br>Qiagen cat. Number: 339306	
miR-210-5p	mmu-miR-210-5p (YP02105697)<br>Qiagen cat. Number: 339306	
Sno202	SNORD68 (mmu) (YP00203911)<br>Qiagen cat. Number: 339306	

Supplemental Table S3. List of antibodies.

Antigen	Supplier	Catalog number
β-Tubulin	Cell Signaling Technology	2146
HIF-1α	Cell Signaling Technology	36169

PDGFRα	R&D Systems	AF1062
OXPHOS	abcam	ab110413
UCP-1	abcam	ab209483
Anti-Mouse IgG	Cell Signaling Technology	7076S
Anti-Rabbit IgG	Cell Signaling Technology	7074S
